# The Role of Myokines in Liver Diseases

**DOI:** 10.3390/ijms26031043

**Published:** 2025-01-25

**Authors:** Hiroki Nishikawa, Soo Ki Kim, Akira Asai

**Affiliations:** 1 Second Department of Internal Medicine, Osaka Medical and Pharmaceutical University, 2-7, Daigakumachi, Takatsuki 569-8686, Osaka, Japan; akira.asai@ompu.ac.jp; 2 Department of Gastroenterology, Kobe Asahi Hospital, Kobe 653-8501, Hyogo, Japan

**Keywords:** myokine, liver, IL-6, sarcopenia, muscle–gut–liver axis, myostatin

## Abstract

Myokine is a general term for hormones, peptides, and other substances secreted by skeletal muscle. Myokine has attracted much attention in recent years as a key substance for understanding the mechanism of “exercise and health”. Skeletal muscle accounts for about 40% of the total human weight and is now recognized as an endocrine organ that produces myokines, which have physiological activity. Representative myokines include IL-6, myostatin, irisin, brain-derived neurotropic factor, fibroblast growth factor-21, and decorin. On the other hand, sarcopenia, defined by quantitative and qualitative loss of skeletal muscle, is a condition that has received much attention in recent years because of its close correlation with prognosis. In patients with chronic liver disease (CLD), sarcopenia is a common complication. Mechanisms underlying sarcopenia in CLD patients have been reported to involve protein-energy malnutrition, which is characteristic of patients with cirrhosis, signaling involved in protein synthesis and degradation, myokines such as myostatin and decorin, the ubiquitin-proteasome pathway, sex hormones such as testosterone, dysbiosis, and insulin resistance, etc., in addition to aging. Each of these pathological conditions is thought to be intricately related to each other, leading to sarcopenia. This review will summarize the relationship between CLD and myokines.

## 1. Exercise and Myokines

Adenosine tri-phosphate (ATP) is used for muscle contraction during exercise. Muscle contraction is performed using the energy produced when one phosphate leaves ATP and is broken down into adenosine diphosphate (ADP). Since the amount of ATP stored in muscle is very limited, resynthesis and production of ATP are necessary for continued exercise [1]. Skeletal muscle can be viewed as the largest organ, accounting for about 40% of the total weight of the human body, and it has endocrine capacity, if not to the extent of classical endocrine organs such as the pituitary gland, thyroid gland, and adrenal glands [2]. Skeletal muscle is now recognized as a secretory organ that produces a bioactive protein called myokine [3]. Myokine is a word coined from the Greek myo (muscle) and kine (agonist). Secreted myokines act on various organs to regulate their functions through autocrine, paracrine, and endocrine pathways. Many myokines are stimulated by muscle contraction and are closely involved in the acute response to exercise and adaptation to habitual exercise. Factors that increase in the blood due to exercise are now also collectively referred to as “exerkines” [4]. On the other hand, some myokines are decreased by inactivity, aging, and high-fat diets, while others are increased, suggesting that these changes affect the risk of disease exacerbation and age-related functional decline [5,6], attracting attention as target factors in the prevention of sarcopenia and frailty. According to proteomic and secretome analyses, skeletal muscle has been reported to secrete more than 100 proteins and peptides [7]. Lactic acid and amino acids secreted from muscle cells [8], microRNAs, and nucleic acids such as mitochondrial DNA [9,10] can also be considered myokines in a broad sense. It was also found that some myokines are packed in exosomes (membrane-bound extracellular vesicles) and secreted out of cells [11], and further functional myokines are expected to be identified in the future.

Exercise increases various cytokines in the blood [12,13], and since IL-6 is the fastest and most abundant to be mobilized into the blood [14], many studies on IL-6 in relation to exercise have been conducted. Initially, the increase in IL-6 in peripheral blood after exercise was thought to be due to inflammation induced by intense exercise. However, Pedersen et al. demonstrated that IL-6 is produced and secreted from contracting skeletal muscle during exercise and that IL-6 is mobilized into the blood without an increase in the inflammatory cytokine TNF-α [13], showing for the first time that skeletal muscle has a role as an endocrine organ. Subsequently, many myokines have been implicated in the regulation of nutrient metabolism in skeletal muscle. Many myokines, including IL-6 secreted by exercise, contribute to the metabolism of carbohydrates and fats in skeletal muscle itself, liver, and adipose tissue [15]. There are myokines with various characteristics, such as myokines that are secreted by a single exercise and contribute to energy supply and blood glucose maintenance during exercise and myokines that contribute to metabolic adaptation by training. On the other hand, many myokines are also involved in muscle atrophy and metabolic disorders associated with inactivity and aging [16]. In addition, some have been reported to contribute to the browning of white adipocytes (making them easier to expend energy), bone metabolism, central nervous system function, and regulation of skin conditions [15]. While there are molecules whose secretion is decreased by muscle contraction. Macrophage migration inhibitory factor (MIF) was discovered as a factor that keeps macrophages in the inflamed area during inflammation [17]. MIF secretion is inhibited during muscle contraction. Insulin acting on skeletal muscle significantly increased glucose uptake in the muscle, but when insulin was added after MIF was applied to skeletal muscle, glucose uptake was significantly suppressed compared to muscle without MIF [18].

In recent years, studies have evaluated myokine kinetics in elderly subjects with exercise intervention. It has been reported that exercise lowers myostatin, which promotes muscle atrophy, and increases follistatin and irisin, which enhance protein synthesis and myocyte formation [19,20]. It has also been reported that insulin-like growth factor-1 (IGF-1), which activates muscle protein synthesis, has lower blood levels in the sarcopenia group than in the non-sarcopenia group, and that there is an association between muscle mass and muscle strength and IGF-1 [21]. Advances in the treatment of sarcopenia using these myokines are expected and are interesting from the perspective of drug discovery. Representative myokines and their roles are listed in Table 1.

## 2. Sarcopenia and Myokines in Liver Disease

**Figure 1:**
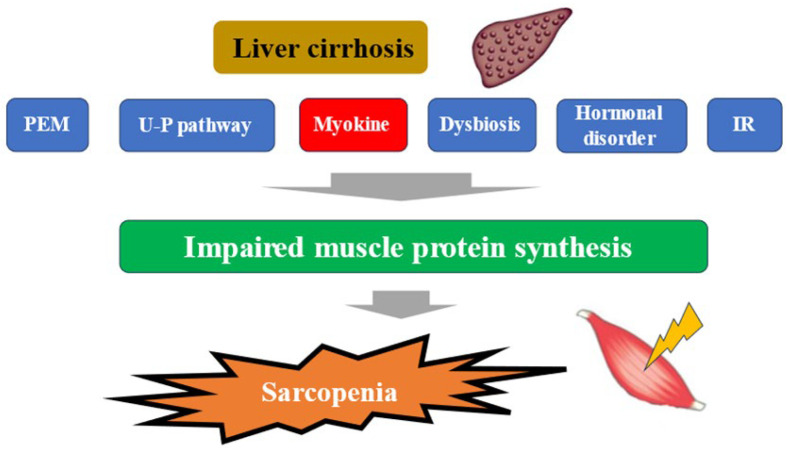
Pathogenesis of cirrhosis leading to sarcopenia. PEM; protein-energy malnutrition, U-P; ubiquitin-proteasome, IR; insulin resistance.

The liver plays a central role in nutrient metabolism and storage. In the energy metabolism, it is closely involved in glycogen metabolism, glycogenesis, and ketone body production from fatty acid degradation, while in the protein metabolism, it is closely involved in protein synthesis and degradation [22]. In Japan, patients with liver disease are aging significantly, and attention should be paid to the increase in the number of patients with liver disease associated with sarcopenia in the future [23]. In a large study (4811 cases) of elderly Japanese with independent daily living, the prevalence of sarcopenia was shown to be around 7% [24]. The complication rate of sarcopenia in cirrhotic patients is reported to be 30–70%. This seems to be high considering that the complication rate of sarcopenia in inflammatory bowel disease, a typical disease of sarcopenia secondary to pathological conditions, is about 20% [25,26,27]. In Japanese patients with cirrhosis, the annual rate of skeletal muscle mass (SMM) loss is 1.3% in Child-Pugh A, 3.5% in Child-Pugh B, and 6.1% in Child-Pugh C, indicating that muscle loss becomes more pronounced as liver function worsens, which is clearly higher than the 1% annual rate of muscle loss in the average elderly person. This may strongly reflect the pathogenesis of secondary sarcopenia in the context of cirrhosis [28]. More recently, a meta-analysis from overseas has shown that (1) the complication rate of sarcopenia in alcoholic cirrhosis is significantly higher than in non-alcoholic cirrhosis (49.6% vs. 33.4%); (2) the sarcopenia complication rates for Child-Pugh A, B, and C cirrhosis are 28.3%, 37.9%, and 46.7%, respectively; (3) the 5-year survival rates for sarcopenic cirrhosis and non-sarcopenic cirrhosis are 45.3% and 74.2%, which indicates a large gap,; in other words, sarcopenia was reported to be a strong prognostic factor in patients with cirrhosis [27]. Mechanisms underlying sarcopenia in patients with liver disease have been reported to involve protein-energy malnutrition, which is characteristic of patients with cirrhosis, signaling involved in protein synthesis and degradation, myokines such as IGF-1, myostatin, and decorin, the ubiquitin-proteasome pathway, sex hormones such as testosterone, dysbiosis, insulin resistance, etc., in addition to aging [29,30,31,32] (Figure 1). In liver disease, each of these pathological conditions is thought to be intricately related to each other, resulting in sarcopenia. The following sections will outline the relationship between myokines and pathological conditions in liver diseases, the Muscle–Gut–Liver axis, and perspectives for drug discovery.

## 3. MASLD, Liver Fibrosis, and Myokines

Recently, non-alcoholic fatty liver disease (NAFLD) has been renamed metabolic dysfunction associated steatotic liver disease (MASLD). The reason for the name change is that “alcoholic” and “fatty” are considered inappropriate terms [33]. Although various pathological conditions, such as steatosis and inflammation, are involved in the progression of NAFLD, “liver fibrosis” has been shown to be the main prognostic pathological finding in patients with NAFLD [34]. Large-scale studies in Western countries have reported that the development of liver fibrosis is associated with hepatocarcinogenesis. Recently, Fujii et al. showed in a nationwide multicenter study using histological findings by liver biopsy that liver fibrosis is also associated with all-cause mortality and liver disease-related mortality in 1398 Japanese NAFLD patients [35]. Furthermore, liver fibrosis has been reported to be involved in the development of cardiovascular disease, the main cause of death in NAFLD [36]. In addition, it has been reported that the development of liver fibrosis is associated with decreased social productivity through worsening patient reported outcomes (PROs) such as fatigue [37]. Thus, liver fibrosis is an important therapeutic target in NAFLD/non-alcoholic steatohepatitis (NASH), which is associated with severe adverse events, poor prognosis, and reduced PROs. Our previous study in patients with metabolic dysfunction associated fatty liver disease also showed a significant correlation between an elevated FIB4 index and the percentage of cases with SMM loss (in our data, for men, the proportion of subjects with SMM loss stratified by FIB4 index was 11.0% (154/1407) in subjects with FIB4 index < 1.30, 24.0% (135/563) in subjects with 1.30 < FIB4 index < 2.67, and 18.2% (8/44) in subjects with FIB4 index > 2.67 (overall *p* < 0.0001). For women, the proportion of subjects with SMM stratified by FIB4 index was 9.7% (66/683) in subjects with FIB4 index < 1.30, 18.1% (47/260) in subjects with 1.30 < FIB4 index < 2.67, and 50.0% (3/6) in subjects with FIB4 index > 2.67 (overall *p* < 0.0001)) [38].

**Figure 2:**
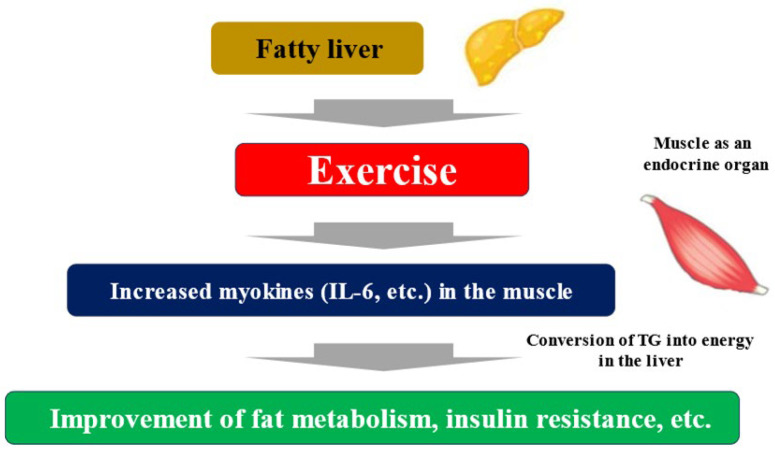
Fatty liver, exercise, and myokines. TG; triglyceride.

Exercise and nutritional therapy are the first-line treatments for MASLD [33]. The endocrine function of muscle has been focused on as a mechanism by which exercise improves liver fibrosis associated with MASLD. Myokines secreted by muscle tissue due to muscle contraction interact with multiple organs, including the liver [39]. Some myokines induce an anti-inflammatory response with each exercise, and long-term exercise is thought to improve cardiovascular risk. Furthermore, myokine has been shown to have a wide variety of systemic effects, including glycogenesis in the liver, glucagon-like peptide-1 (GLP-1) expression in the intestinal tract, cognitive function, fat and glucose metabolism, browning of white adipocytes, bone formation, endothelial cell function, skin structure, and tumor growth [40]. Representative myokines include IL-6, IGF-1, myostatin, irisin, brain-derived neurotropic factor (BDNF), fibroblast growth factor (FGF)-21, and decorin. Muscle strength training causes myokines such as IL-6 to be secreted into the blood from the muscles. When these myokines reach the liver, they promote the ability of hepatocytes to convert triglyceride into energy. As a result, triglycerides stored in the liver are reduced, and fatty liver improves (Figure 2) [41]. Exercise can result in weight loss and improved systemic metabolism, but a 30 min walk burns a mere 100 kcal. Rather than the effect of calorie consumption, the improvement in the systemic metabolism through muscle movement is considered to be more significant. It is known that the amount of myokines secreted by muscles is almost the same for both high- and low-burden exercises [42]. Exercise itself causes significant metabolic changes in muscle, and one mechanism other than myokines is the activation of AMP activated protein kinase (AMPK) and its downstream molecules, which improves insulin resistance, etc. [43]. AMPK is a major regulator of glucose and fat metabolism in skeletal muscle and has been reported to be activated by exercise and other interventions such as low-energy diets and food-derived functional ingredients, contributing to improved endurance exercise capacity, obesity, and glucose tolerance [44].

Myokine, FGF-21, has the effect of increasing brown adipose tissue thermogenesis, which breaks down fat in the liver and improves fatty liver [45]. Irisin regulates the metabolism of glucose and fats in adipose tissue and also increases energy expenditure and improves fatty liver by promoting brown adipogenesis of white adipocytes [46]. BDNF plays an important role in learning and memory functions by maintaining neuronal survival, promoting neurite outgrowth, and promoting neurotransmitter synthesis. Depression involves decreased BDNF expression in the hippocampus, and it has been reported that antidepressants increase BDNF and improve depressive symptoms [47]. In humans, BDNF-mediated improvement of depression by exercise has also been demonstrated. Furthermore, BDNF has been reported to inhibit eating behavior and improve glucose metabolism. Several reports have shown that transient exercise increases BDNF expression in skeletal muscle, but some reports have shown no BDNF increase in blood [48]. Interestingly, BDNF-deficient mice develop NASH [49]. Leptin is secreted by adipocytes and produces potent feeding inhibition and increased energy expenditure, mainly through receptors in the hypothalamus. It has been reported that human skeletal muscle also produces leptin [50]. Deficient leptin action is thought to play an important role in the pathogenesis of obese MASLD. Several reports have shown that exercise improves leptin resistance [51].

## 4. Liver Cirrhosis and Myostatin

Clinical features such as gynecomastia associated with a decrease in the sex hormone androgen and encephalopathy associated with a decrease in ammonia clearance due to urea circuit dysfunction are observed in cirrhotic patients. Low serum androgen levels and high serum ammonia levels directly inhibit muscle protein synthesis and induce myostatin (described later) [23]. For this reason, sarcopenia complication rates are high in liver disease, and liver disease is representative of secondary sarcopenia.

Myostatin was discovered as one of the TGF-beta family members that inhibits muscle growth [52]. Myostatin is a myokine that strongly inhibits the synthesis of muscle proteins and maintains muscle homeostasis in the human body through its joint action with myokines that promote muscle protein synthesis [53]. In our study of 198 patients with cirrhosis, for the entire cohort, the 1-, 3-, and 5-year cumulative overall survival (OS) rates were 93.9%, 72.7%, and 50.4%, respectively, in the higher myostatin group, and 97.0%, 83.3%, and 73.6%, respectively, in the lower myostatin group (*p =* 0.0001). We also found that serum myostatin level was significantly higher in patients with Child-Pugh B or C cirrhosis than in those with Child-Pugh A cirrhosis, indicating that the synthesis of muscle protein is more suppressed, or more likely to develop sarcopenia, as liver function worsens [30]. Cirrhotic patients are prone to hyperammonemia due to decreased ammonia clearance, as mentioned earlier. In our study, serum ammonia levels showed a significant positive correlation with serum myostatin levels [30]. Similar findings have been reported from overseas in recent years [54]. Our further study also showed that serum myostatin levels were significantly inversely correlated with serum zinc levels, suggesting an association between trace element deficiency and SMM loss in liver cirrhosis.

## 5. Hepatocellular Carcinoma (HCC) and Myokines

Decorin antagonizes myostatin and promotes muscle hypertrophy when blood levels are increased by exercise [55]. Kawaguchi et al. measured decorin in 65 HCC patients indicated for transcatheter arterial chemoembolization therapy and divided them into two groups according to the median value of decorin: high-decorin group and low-decorin group, and examined physical function and prognosis. The 6 min walking distance in the high-decorin group was significantly longer than that in the low-decorin group. In addition, the low-decorin group had a median survival of 463 days, whereas the high-decorin group had a significantly prolonged survival of 732 days [56]. Decorin may be closely related to physical function and prognosis in patients with HCC. Yoshio et al. investigated serum myostatin levels in patients with HCC who underwent hepatic resection with a diagnosis of solitary HCC and reported that serum myostatin levels were higher as liver fibrosis progressed [57]. They also reported that high myostatin levels are a poor prognostic factor [57]. They also confirmed the fact that liver fibroblasts are activated by myostatin stimulation and produce collagen [57]. Choi et al. used serum from 238 HCC patients to measure three myokines: myostatin (median value: 3979.3 for male and 2976.3 pg/mL for female), follistatin (median value: 2118.5 for male and 2174.6 pg/mL for female), and IL-6 (median value: 2.5 for male and 2.7 pg/mL for female). All three myokines were measured higher than in healthy subjects, and they reported that the serum follistatin level was an independent factor for poorer OS in HCC patients [58].

## 6. Effects of Myokine on Enterohepatic Circulation

The metabolic capacity of skeletal muscle is affected by many hormones, cytokines, and metabolites derived from other organs. Many of these are increased in the blood by single or habitual exercise and decreased by inactivity and aging. On the other hand, the intestine plays an important role in defense and immunity by digesting and absorbing nutrients and controlling the entry of foreign substances. The tight junctions between intestinal epithelial cells act as a barrier to prevent the entry of bacteria, endotoxins, antigens, etc. When intestinal barrier function is compromised, they may enter the circulation and elicit an inflammatory response. As a result, it has been observed in a mouse model of increased intestinal permeability that it deteriorates muscle function, such as decreasing insulin sensitivity and mitochondrial activity in skeletal muscle [59]. In mice with increased intestinal permeability, there is an increase in blood lipopolysaccharide (LPS) concentration as well as a decrease in glycogen stores and pH in muscle tissue [59]. Short-chain fatty acids are known to act directly or indirectly on skeletal muscle, increasing glucose uptake into muscle, inhibiting muscle protein degradation, and contributing to anti-inflammation and metabolic improvement [60]. It has been suggested that some of the lactic acid produced by exercise is metabolized by intestinal bacteria (e.g., *Veillonella* spp.) into propionic acid, which supports muscle endurance [59]. Bile acids increase the uptake of glucose into muscle and promote muscle protein synthesis [61]. Thus, there is a close linkage between gut homeostasis and skeletal muscle homeostasis. Skeletal muscle weakness associated with aging and cachexia has also been implicated in reduced gut barrier function [62]. LPS and indoxyl sulfate derived from dysbiosis activate proteolytic signals, promote muscle atrophy and liver steatosis, and exacerbate glucose metabolism. Therefore, the maintenance of the intestinal barrier contributes to the prevention of metabolic syndrome, sarcopenia, and frailty by regulating blood glucose levels, body fat mass, muscle mass, etc. [63]. A high rate of increased intestinal permeability is observed in cirrhosis, which suggests that cirrhosis is more likely to be complicated by sarcopenia or frailty [32].

**Figure 3:**
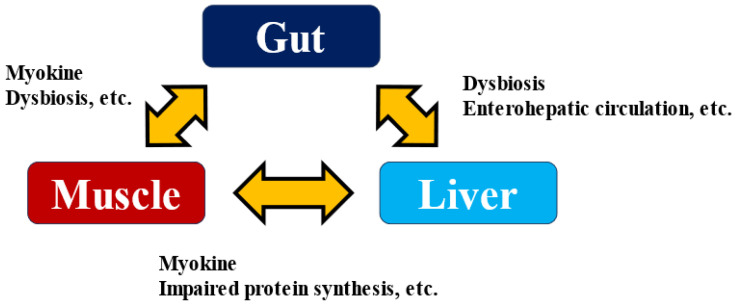
Muscle–Gut–Liver Axis.

Several myokines have been suggested to affect gut function. The intestine is an organ closely associated with immune function, as most immune cells are concentrated in the intestine. For example, IL-6 and IL-15 act on neutrophils, increasing the production of anti-inflammatory factors such as IL-10 and IL-1 receptor agonists and promoting lymphocyte and natural killer cell proliferation and tissue infiltration [64]. It has also been reported that IL-6 promotes GLP-1 secretion from enteroendocrine L cells [65], and the effect on insulin secretion and sensitivity via enterohepatic circulation may be suggested. Enterohepatic circulation is a cycle in which biological substances and drugs are secreted into the duodenum via the bile duct along with bile, absorbed again from the intestinal tract, and returned to the liver via the portal vein. On the other hand, it has been shown that intestinal metabolites via enterohepatic circulation play a very significant role in the development and exacerbation of liver diseases [66,67]. Excessive alcohol consumption and a high-fat diet disrupt the intestinal barrier. They affect not only the composition of the gut bacteria but also their metabolites and the microbe-associated molecular patterns (MAMPs) involved in their interactions with the host, which in turn strengthens the influence of the bacteria themselves on the liver. As a result, it promotes the development of hepatitis, liver fibrosis, cirrhosis, and even HCC [68]. When the intestinal barrier is breached by various stresses, LPS and lipoteichoic acid induce inflammatory signals via Toll-like receptors in the liver, promoting liver fibrosis and HCC. Skeletal muscle weakness reduced intestinal barrier function, and the onset and exacerbation of liver disease are closely related. Elucidating the mechanism of liver disease pathogenesis via the Muscle–Gut–Liver Axis (Figure 3) [69,70] will lead to the development of methods to prevent the development of liver disease, and myokine research will play a role in this process.

## 7. Myokine as a Potential Drug Discovery: Myostatin Inhibitor

As mentioned above, myostatin has attracted attention as a myokine that acts specifically on skeletal muscle. When it was first discovered, the term myokine was not commonly used, and it attracted attention as a molecule that plays an important role in the regulation of SMM because it is expressed and secreted in skeletal muscle, and myostatin-deficient mice show a marked increase in muscle mass [52]. Myostatin is secreted from skeletal muscle and retained in a latent form. When the N-terminal propeptide is cleaved by metalloproteinases, it becomes the mature form and transmits signals into the cell via activin receptor type IIB to negatively regulate muscle growth. The function of myostatin can be inhibited in the blood by binding to follistatin [71]. The correlation between serum myostatin levels and muscle mass [72] and reports of elevated myostatin levels in the elderly [73] suggest that myostatin inhibitors may be effective in preventing muscle atrophy in actual clinical practice. In recent years, drug discovery research on muscle atrophy inhibitors targeting myostatin receptors such as activin receptor IIB and follistatin has attracted much attention. It has been reported in animal studies that myostatin inhibitors increase muscle mass in various models of muscle atrophy [74,75], making them very interesting as a treatment for muscle atrophy. Myostatin inhibitors, with their efficacy and low side effect potential, are promising as treatments for cancer cachexia, cirrhosis, skeletal muscle atrophy such as sarcopenia in the elderly, and neuromuscular diseases such as progressive muscular dystrophy [76]. Myostatin inhibitors have also been reported to inhibit adipocyte hypertrophy and have the potential to be successful in the treatment of obese MASLD [77].

In recent years, there has been growing interest in combination therapy with GLP-1 receptor agonists and myostatin inhibitors in the treatment of obesity. Since GLP-1 receptor agonists decrease muscle as well as fat, it is hoped that the combination of myostatin inhibitors may reduce SMM loss [78]. Myostatin inhibitory peptides are easily transferred to muscle tissue and also inhibit myostatin produced in muscle tissue [78].

## 8. Closing Remarks

The role of myokine, which has been the focus of much attention in recent years, particularly in liver disease, was outlined in this article. Pedersen et al. discovered the role of IL-6 as a myokine, and since then many myokines have been identified, demonstrating that myokines have various effects on various organs throughout the body. The liver plays a central role in nutrient metabolism and storage. The concept of Muscle–Gut–Liver axis is very important. In considering the prevention of the onset and progression of liver disease, it is important to take an organ network. The progress of the drug discovery will also be a great blessing for patients with liver disease, which is associated with a high rate of sarcopenia.

## Figures and Tables

**Table 1:** Representative myokines.

Myokines	The Role in the Human Body
IL-6	It is released into the blood from contracted skeletal muscle during exercise and promotes glucose uptake in skeletal muscle and glucose release in the liver.
Myostatin	Myokines that inhibit muscle hypertrophy. Exercise suppresses myostatin secretion.
IGF-1	Influences muscle satellite cells, muscle protein synthesis and degradation, and gene expression to promote muscle hypertrophy
Follistatin	Secreted proteins that bind to TGF-β family members such as myostatin and modulate their activity by inhibiting their binding to the receptor.
Irrisin	It improves weight loss and insulin resistance by significantly increasing systemic energy expenditure. It also plays an important role in the regulation of fatty acid beta-oxidation and fat metabolism in the liver.
BDNF	An autocrine or paracrine myokine widely expressed in the adult brain, and it acts on peripheral tissue metabolism, including fatty acid oxidation, and reduces adipose tissue mass.
Decorin	It antagonizes myostatin and promotes muscle hypertrophy when blood levels are elevated by exercise.
FGF-21	It increases brown adipose tissue thermogenesis, which breaks down fat in the liver and improves fatty liver.

IGF-1; insulin-like growth factor-1, BDNF; brain-derived neurotropic factor, FGF; fibroblast growth factor.

## Abbreviations

ATP: adenosine tri-phosphate; ADP: adenosine di-phosphate; MIF: macrophage migration inhibitory factor; IGF-1: Insulin-like growth factor-1; NAFLD: non-alcoholic fatty liver disease; MASLD: metabolic dysfunction associated steatotic liver disease; PRO: patient reported outcome; NASH: non-alcoholic steatohepatitis; SMM: skeletal muscle mass; GLP-1: Glucagon-like peptide-1; BDNF: brain-derived neurotropic factor; FGF: fibroblast growth factor; AMPK: AMP activated protein kinase; OS: overall survival; HCC: hepatocellular carcinoma; LPS: lipopolysaccharide.
